# Possible viral infections in flood disasters: a review considering 2019 spring floods in Iran

**Published:** 2019-04

**Authors:** Jila Yavarian, Nazanin Zahra Shafiei-Jandaghi, Talat Mokhtari-Azad

**Affiliations:** Department of Virology, School of Public Health, Tehran University of Medical Sciences, Tehran, Iran

**Keywords:** Viral infections, Flood, Disaster

## Abstract

Floods are one of the natural disasters occurring worldwide which have a massive range of health impacts. In addition to immediate dangers such as drowning, floods can increase the transmission of some communicable diseases. Up to now there was no report of viral infection outbreaks after 2019 spring floods in Iran. This review explains the possible viral infections which may occur during or after floods.

## INTRODUCTION

Floods are common disasters worldwide. In 2016, globally more than 74 million people were affected by flooding, lead to 4720 deaths with high economic cost, which 43% were happened in Asia (1–3). Floods occurrence has tended to increase in recent decades, and this trend is likely to enhance with climate change. According to the Iranian Department of Environment, of 421 flood events from 1951 to 2001 nearly 74% were only from 1981 to 2001 which during this period the flood rate was 20 times higher than before. Since 2001 to 2019, there were several floods in different parts of the country, which the last one was in spring 2019. In fact, during March and April 2019, Iran experienced flooding in three provinces (Khuzestan, Lorestan and Golestan) which affected many people that were displaced and moved into the camps. These floods had adverse impacts on living conditions in affected areas.

Health effects often related to floods are gastrointestinal and respiratory infections which are major reasons of diseases and death in people displaced by natural disasters. Lack of access to health-care services and crowding, augment the risk of death from these infections. Floods negatively affect supply systems and water sources, in addition to waste-disposal systems and sewerage, then the transmission of pathogens is prone to be increased (4).

**Fig. 1.**
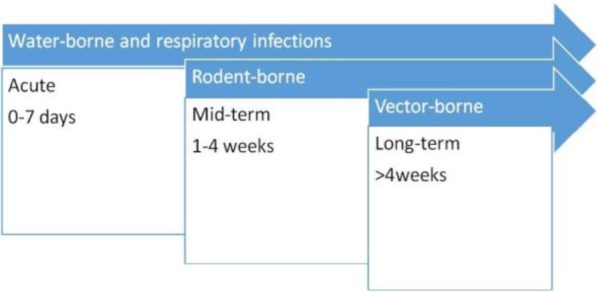
The occurrence of diseases outbreaks after flood disasters in relation to time.

The risk of viral infections after flood is really important health issue and different classes of infectious diseases can cause outbreaks during the weeks after flooding as shown in Fig. 1. Floods can increase the transmission of viral diseases specially water born infections, such as diarrheal diseases, hepatitis A and E, air-borne infections and vector borne diseases such as yellow fever, west Nile fever (WNF) and dengue fever (5, 6). This review explains the viral infections during and after floods.

### Respiratory infections.

Respiratory tract infections (RTIs) are one of the most commonly reported infections after flooding due to overcrowding in shelters. After 1988 devastating floods in Bangladesh, RTIs were reported 17.4% of all diseases and 13% of death reasons (7), similarly, 113981 cases of RTIs were reported in Pakistan after floods (8).

Fortunately two months after 2019 flooding in Iran, there was no report of respiratory infection outbreaks. However based on the previous studies, there is still a potential risk for it. Possible viral respiratory pathogens which might cause RTI outbreaks in survivors affected by recent floods could be predictable based on the annual epidemics and history of viruses’ circulation. In early spring in Iran, influenza viruses and respiratory syncytial virus circulations were reported in previous years in Khuzestan and Golestan (9–11). Among other viral causes of RTI, adenoviruses are common in the late winter, spring, and early summer, consequently adenoviruses might circulate in affected population. However the risk of viral RTI outbreaks would decrease in summer and again would increase in autumn and winter.

### Gastrointestinal infections.

Due to the problems in sewage systems, poor hygiene, overcrowding and unhealthy drinking water, viral gastroenteritis may occur. After hurricane Katrina, outbreaks of norovirus gastroenteritis were reported (12). There was an outbreak of norovirus in American tourists in Germany who had exposure to flood water polluted with raw sewage (13). In the Solomon Islands, after flash flooding, rotavirus caused diarrheal illnesses (14). In Dhaka, Bangladesh, after cholera, rotavirus was the second frequently identified pathogen in flood-associated diarrheal epidemics (15). Ingestion of contaminated water or food, is the main route for transmission of hepatitis A and E, as reported in India and Sudan (16, 17). Shears et al. reported the increased number of hepatitis A infections after Khartoum floods in 1988 (18). In Italy the number of hepatitis A cases was increased in Liguria, Piemonte and Lombardia between 2000 and 2003 during flood disasters in 2000 and 2002 (19). Setzer et al. found a considerable increase in outpatient visits for enteric adenoviruses after Hurricane Floyd in affected areas, in comparison with non-affected counties (20). There was not any report of gastrointestinal disease outbreak after different floods in Iran, but in general outbreaks of hepatitis A and E and enteroviruses might happen in areas affected by flooding.

### Mosquito borne diseases.

After flooding, mosquito borne diseases for example dengue fever, especially in endemic areas, can increase. Standing water after overflow of the rivers could act as the breeding sites for mosquitos. Therefore vector borne diseases have long-term impacts on public health (Fig. 1). In the flooding in northern Peru in 2017, outbreaks of chikungunya and dengue fever with more than 19000 suspected cases of dengue fever were reported (21). Outbreaks of WNF after flooding occurred in Italy in 1998, Czech Republic in 1997 and Romania in 1996–97 (22). In a flood in Punjab/Pakistan in 2010, 21204 people were reported with dengue fever (23, 24). Again in 2011–2013 dengue infections were reported after flooding which in Karachi, 700, 858, 630 and 2700 deaths related to dengue fever were identified in 2010, 2011, 2012 and 2013 respectively (25). Rift Valley fever (RVF) is another mosquito borne disease with the possibility of causing outbreak after flooding. Many studies in Kenya reported outbreaks of RVF after high rainfall and flooding (26). Anyamba et al. reported RVF in late 1957, 1982 and mid 1989 after a rainfall in Kenya (27). The largest outbreak of RVF happened after El Nino flood in 1997/98 in Kenya which lead to 89000 infections with 0.5 % mortality (28). In 2006, in another flooding in Kenya, RVF outbreaks were reported (29). In 2007 in Sudan, RVF outbreak occurred with 747 confirmed cases including 230 deaths (mortality rate 30.8%) (30). In a study by Hubalek Z et al. in Czech Republic, specimens from people affected by the 2002 flood were tested serologically for mosquito borne viruses. Antibodies were detected against Batai (0.2%), Sindbis (1%), and Tahyna (16%) viruses, but not WNV. Paired serum samples showed one Tahyna bunyavirus infection (31). A study in Brisbane, Australia showed that increases in the high tide and rainfall in one month were considerably associated with the rise of monthly Ross River virus incidence (32). Crimean-Congo hemorrhagic fever (CCHF) is endemicin 23 of 30 provinces of Iran; Sistan-Baluchestan, Isfahan, Fars, and Khuzestan are the most highly infected provinces respectively. In a study by Sharififard et al. two peaks of CCHF infections reported in Khuzestan, during 1999 - 2015 (in 2003 and 2010) (33). In another study by Sedaghat et al. CCHFV was found in 5.3% of the ticks collected in Golestan province during 2014–15 (34). Pourmahdi Borujeni et al. showed the existence of WNV infection in Khuzestan (35). In a research in Mahshahr strict, Khuzestan, some vectors of human and animal pathogens were identified (36). A study (2008–2014) has reported *Aedes albopictus* in southern Iran (37). The species *A. albopictus* is well-known for transmitting chikungunya and dengue viruses. In another research, *Aedes unilineatus* was also recognized in the southeast of Iran (2012–2014). This mosquito species has been identified as a dengue vector in Karachi, Pakistan (38). These findings support establishment of dengue vectors in this region. Based on the different distribution of vectors, there is the possibility of re-emergence of related diseases in Khuzestan and Golestan provinces after flooding.

### Rodent borne diseases.

Rodent borne diseases also increase during massive rainfall and flooding. In Mazandaran, Gilan and Golestan provinces, distribution of some rodents were identified including: *Rattus (R.) norvegicus*, *R. rattus*, *Apodemus sylvaticus*, Arvicola, Mus musculus, Nesokiaindica, *Cricetulus migrates* and *Rhombomys opimus* (39). Hantaan, Puumala, Dobrava, Seoul and Nairoviruses are rodent borne viruses which have been recognized in Iran, then the outbreaks of these viruses after flooding might occur (40).

## CONCLUSION

In conclusion, this review presented the significance of viral infections in flood disasters. Public health interventions before, during and after floods need to take place to decrease the risk of infectious diseases. At the first week of the flooding, a rapid risk assessment must be performed and data should be collected from flooding areas, people and the important disease threats for identification of adequate interventions. Sanitation, hygiene, nutrition, water and shelter providing are the immediate public health response in flooding but there must be a plan for decreasing morbidity and mortality related to flood disasters. According to the local weather conditions, floods and the facility of vector-borne virus transmission, the authorities should take measures to control the infection, for instance as Hyalomma ticks are the main vectors of CCHFV in Golestan Province, preventive strategies using acaricides and repellents to avoid contact with Hyalomma ticks are suggested.

Before or even after floods, the government should improve vaccination programs for preventable viral diseases and vector and rodent control programs in areas susceptible to natural disasters. These arrangements can be effective in decreasing the burden of viral diseases.
